# Heavy-atom effect on optically excited triplet state kinetics

**DOI:** 10.1371/journal.pone.0184239

**Published:** 2017-11-20

**Authors:** Christian Hintze, Tobias O. Morgen, Malte Drescher

**Affiliations:** Fachbereich Chemie, Universität Konstanz, Konstanz, Germany; Martin-Luther-Universitat Halle-Wittenberg, GERMANY

## Abstract

In several fields of research, like e.g. photosensitization, photovoltaics, organic electroluminescent devices, dynamic nuclear polarization, or pulsed dipolar electron paramagnetic resonance spectroscopy, triplet state kinetics play an important role. It is therefore desirable to tailor the kinetics of photoexcited triplet states, e.g. by exploiting the intramolecular heavy-atom effect, and to determine the respective kinetic parameters. In this work, we set out to systematically investigate the photoexcited triplet state kinetics of a series of haloanthracenes by time-resolved electron paramagnetic resonance spectroscopy in combination with synchronized laser excitation. For this purpose, a procedure to simulate time traces by solving the differential equation system governing the triplet kinetics numerically is developed. This way, spin lattice relaxation rates and zero-field triplet life times are obtained concurrently by a global fit to experimental data measured at three different cryogenic temperatures.

## Introduction

Photoexcited triplet states found numerous applications in several fields of research like, for example, photovoltaics [1], photosensitization [2], photon upconversion at low light intensities via triplet-triplet annihilation [3], and organic electroluminescent devices [4]. In nuclear magnetic resonance (NMR) spectroscopy, dynamic nuclear polarization (DNP) by photoexcited triplet states is used to increase its sensitivity. [5] Here, utilizing the much higher polarization of optically excited triplet electron spins [6] ^1^H NMR spin polarizations of 70% and more were achieved. [7, 8] In another recent development, the triplet state was successfully employed for distance measurements in the nanometer range by triplet-doublet DEER and LaserIMD. [9–12]

In the aforementioned applications, the kinetic behavior of the triplet system chosen might be a limiting factor. If so, one would like to tailor the chromophore used to enhance its utility in its application. For example, in order to achieve high polarization transfer in DNP, the triplet spin polarization has to persist for long times. In LaserIMD, a slow spin-lattice relaxation (SLR) is needed as well to avoid dampening of the dipolar modulation. In triplet-doublet DEER, fast SLR impacts data quality negatively. At the same time, in those examples, the excited triplet state should decay to the ground state fast enough, such that enough chromophores are available for repeated excitation after the experimental repetition time.

An easy way to change the excited triplet state kinetic properties of a chromophore via chemical modification is exploiting the intramolecular heavy-atom effect. This effect has been investigated extensively on anthracene and, specifically, on haloanthracenes. [13] In this work we set out to systematically investigate the photoexcited triplet state kinetics of a series of haloanthracenes by means of electron paramagnetic resonance (EPR) spectroscopy [14]. We chose anthracene because the photochemistry of anthracene has been studied in detail [15–17], its triplet state is well-known (more than 500 corresponding publications in Thomson Reuters Web of Science) and the intersystem crossing (ISC) of haloanthracenes (among other haloaromatics) is understood [18].

Experiments to record the excited triplet state kinetics are well-known in EPR [14] as well as in optically detected magnetic resonance (ODMR) [19–21]. In this work, transient EPR (TR-EPR) is used to obtain EPR spectra of haloanthracenes, and electron spin echo (ESE) EPR for the actual kinetic measurements.

Several procedures for analyzing EPR triplet kinetics were reported. Usually it is ensured experimentally by the choice of temperature whether triplet decay or SLR dominates the kinetics observed, which simplifies the respective analysis of the data. [14] If the choice of temperature is restricted by e.g. by the desired application of the triplet system, triplet decay and SLR might not be disentangled in the kinetic data. For such intermediate cases, an approximate solution has been developed analytically [22] or found numerically by Runge-Kutta methods [23, 24]. In the current manuscript we show that the exact differential equation system can be solved numerically to yield triplet decay as well as SLR kinetic parameters describing the experimental kinetic data in intermediate temperature regimes.

## Materials and methods

All anthracenes were undeuterated and supplied by MCAT, Konstanz, Germany. The solvent used for all experiments was toluene-*d*_8_, provided by Sigma Aldrich. For UV/Vis absorption spectra of all anthracenes cf. S6 Fig. Toluene is a good solvent for the anthracenes and it forms a transparent, rigid glass in the temperature range chosen in our experiments, which is favorable for light excitation. We chose a deuterated solvent due to the slower transversal electron spin relaxation compared to a non-deuterated one, which increases the signal-to-noise ratio (SNR) in pulsed EPR experiments.

All substances were used as is, without further purification prior to the experiments. The spectrometers used are Elexsys E580 from Bruker. The laser system Excistar XS was manufactured by Coherent. The quartz glass fiber was delivered by Lasertechnik Berlin. The laser power was adjusted to yield approximately 3 mJ per laser pulse at a wavelength of 351 nm (XeF). For the relation of the excitation wavelength used to the UV/Vis absorption spectra of anthracenes cf. S6 Fig.

### Transient EPR

The measurements were performed at a temperature of 20 K in a critically coupled Bruker EN 4118X-MD4 resonator in X band with a microwave power of 2 mW. The concentrations of all anthracenes were chosen to be 5 mM. The spectrometer’s built-in transient recorder and the laser were triggered by an external pulse delay generator with a repetition rate of 10 Hz. For every single of the 350 points of magnetic field strength, the average of five to six transients of 4096 ns length was recorded (1024 points and a time resolution of 4 ns). After collection of all transients for every magnetic field value, this procedure was repeated 4–38 times for averaging to improve SNR. Roughly the first third of the averaged transients is used for baseline correction. The first 200–400 ns of the remaining two thirds are integrated to yield the data point for the respective field strength they were recorded at. For the exact numbers of averages, cf. S1 Table.

### Electron spin echo EPR

The measurements were performed in an overcoupled Bruker ER 4118X-MS3 resonator in X band at temperatures of 50 K, 30 K and 10 K. The microwave power was adjusted to yield a *π* pulse length of 16 ns for the detection of triplet electron spins. The concentrations of all anthracenes were chosen to be 1 mM. The laser was triggered by the spectrometer’s built-in pulse delay generator with a repetition rate of 50 Hz. The pulse sequence used was (laser trigger)—*t*_DAF_—$\left(\frac{\pi }{2}\right)$—*τ*—(*π*)—*τ*—(echo). This sequence was repeated several times for one *t*_DAF_, before *t*_DAF_ is incremented. The number of repetitions at one time point is given by the shots per point and the repetition rate is defined by the shot repetition time. The interpulse delay time *τ* was chosen to 420 ns. The Hahn spin echo amplitude is measured by integrating its transient symmetrically around the maximum over the length of the *π* pulse. A two-step phase cycle was used to offset background intensity when the spectrometer’s transients are not perfect zero-lines off resonance. Several scans of such time resolved along *t*_DAF_ Hahn echo sequences are then averaged to increase SNR. For the exact numbers of averages, cf. S2 Table.

### Simulation of EPR spectra

The TR-EPR spectra were simulated using EasySpin, a Matlab package [25]. In order to determine the ZFS parameters, g-value, and the optical spin polarization (OSP) in terms of triplet sublevel populations (EasySpin parameter “Temperature”), the root mean square deviation (RMSD) between the experimental and a simulated spectrum (using EasySpin function “pepper”) was minimized with a genetic algorithm (“ga”, part of the Global Optimization Toolbox of Matlab). The resulting best-fit relative sublevel populations and ZFS parameters are shown in Table 1. For the full set of parameters, cf. S3 Table. Unresolved hyperfine couplings were taken into account (EasySpin parameter “HStrain”, describing a residual linewidth (FWHM) in the frequency domain in MHz) [26]. The hyperfine structure is not resolved due to the observation of triplet powder spectra and a spectral width orders of magnitudes larger than the hyperfine splittings in anthracene [27]. Additional convolutional broadening in the field domain (EasySpin parameter “lw” or “lwpp”) was not applied.

**Table 1:** Relative sublevel populations due to OSP and ZFS parameters of haloanthracenes determined through best fit simulations of the TR-EPR spectra shown in Fig 3.

	*p*_X_:*p*_Y_:*p*_Z_	*D* MHz	*E* MHz
**1**	0.46:0.52:0.02	2167	-247
**2**	0.37:0.60:0.03	2040	-125
**3**	0.56:0.38:0.06	1844	-43
**4**	0.49:0.51:0.00	2169	-249

### Simulation of EPR kinetic time traces

A home written Matlab script determines the best fit to the kinetic time traces in the following steps:

1. data handling
2. randomized parameter initialization
3. minimizing the deviations between experimental and simulated data in terms of reduced *χ*^2^
4. estimating the error of the parameter estimation process by a Monte Carlo simulation approach

*Data handling* entails processing the experimental raw data. Due to sample bleaching by laser irradiation in the UV, the ESE intensities decrease with increasing measurement time, i.e. number of laser flashes emitted onto the sample. Nonetheless, the ESE intensity is sufficient for several consecutive kinetic time trace measurements on different spectral positions. After the corresponding measurement procedure, the individual intensities of the time traces are not comparable and have to be adjusted. For this purpose, the spectral intensities are read from spectral simulations at the respective magnetic field positions (see below). Additionally, a small offset in intensity is introduced in the measurements by the spectrometer due to instrumental imperfections. Therefore, the data is stretched along the ordinate to yield zero intensity for long times *t*_DAF_, maintaining the initial intensities described before. This is reasonable since even residual Boltzmann populated triplet ESE intensity, though surely present, does not exceed noise levels of our measurements on haloanthracenes **2** to **4**, with **1** being the only exception where Boltzmann populated triplet ESE intensity was observed due to its long triplet life time (see below) and higher signal intensity.

*Randomized parameter initialization* is performed for each of a given number of minimization runs. In the data presented here, this was done 100 times, avoiding misinterpretation of occasional local minima and to assess numerical stability of the minimizations.

*Minimization* is performed globally by built-in Matlab functions for all temperatures and canonical orientations simultaneously, with the triplet life time chosen to be independent of temperature. Here, the triplet life time is the mean life time in terms of the average length that an individual molecule remains in its triplet state. In other words, the triplet life time is a measure of the combined radiative and non-radiative triplet decay times. In the data presented here, we used simulated annealing as implemented in the Matlab function “simulannealbnd” (part of the Global Optimization Toolbox), which allows to set upper and lower bounds for the fitted parameters.

For the actual simulation of a kinetic trace, the full matrix differential equation of populations of all three triplet sublevels is used.

$$\begin{array}{c} \left(\begin{array}{c} {\dot{n}}_{+1}\\ {\dot{n}}_{0}\\ {\dot{n}}_{-1} \end{array})=(\begin{array}{ccc} -w_{1}-k_{+1} & w_{1} & 0\\ w_{1} & -w_{1}-w_{2}-k_{0} & w_{2}\\ 0 & w_{2} & -w_{2}-k_{-1} \end{array})(\begin{array}{c} n_{+1}\\ n_{0}\\ n_{-1} \end{array}\right) \end{array}$$ (1)

**Fig 1:**
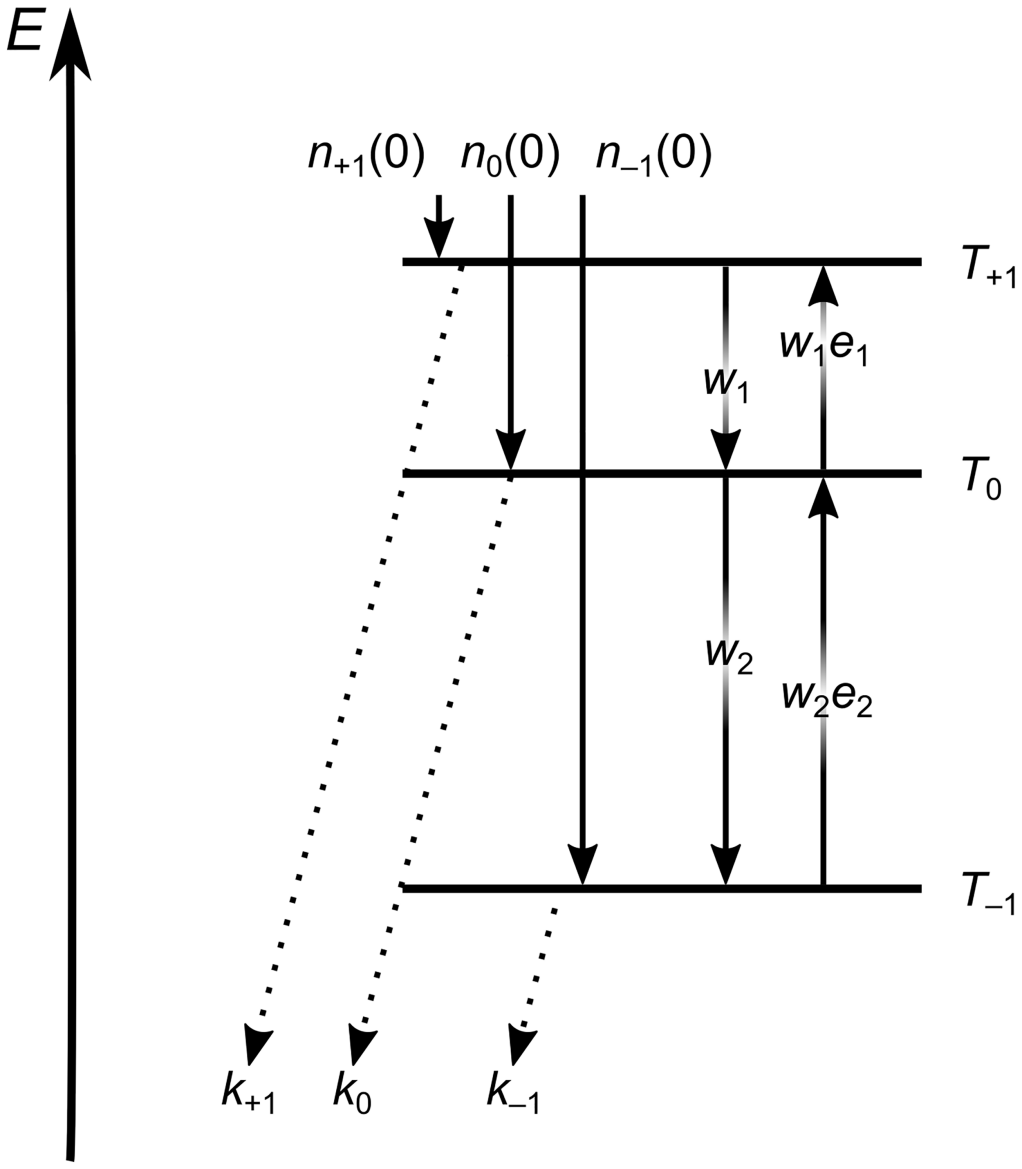
Excited triplet state sublevel scheme and transition rate constants. The dotted arrows indicate combined radiationless (ISC) and radiative (phosphorescence) decay.

Here, the *w*_*i*_ are the SLR rates and the *k*_*j*_ is the triplet decay rate of the triplet sublevel *j*, shown in Fig 1. The actually observed SLR rates *w*_*i*_ are effective rates for all transitions observed on one spectral position of the powder spectrum. The only way to avoid this systematic deviation would be to use crystalline samples to separate the transitions by specifically orienting the triplet molecules. On the other hand, if the kinetic parameters are of interest in a solvent that is not accessible in crystalline form, this cannot be avoided. Another simplification introduced is a high temperature approximation, meaning *w*_*i*_ ≈ *e*_*i*_*w*_*i*_, with the Boltzmann factor *e*_*i*_ approximated as $e_{i}=\exp(-\frac{\Delta E_{i}}{k_{b}T})\approx 1$, which introduces negligible deviation at the temperatures used.

In order to reduce the total number of free parameters in the simulations, the data was fit globally over all temperatures and orientations simultaneously. This way, the high-field triplet life times *k*_*j*_ are related to each other via the zero-field triplet lifte times *k*_X,Y,Z_, e.g. for the Z canonical orientation (the other orientations are given by permutation):

$$k_{+1}=k_{0}=\frac{1}{2}(k_{X}+k_{Y})$$ (2)

$$k_{0}=k_{Z}$$ (3)

The homogeneous differential equation system is diagonalized to yield the eigenvalues and eigenvectors of the corresponding matrix representation given in Eq 1 using the Matlab function “eig”. These are used to solve for the set of fundamental solutions and the kinetic trace is calculated.

Note that three initial values are needed to solve the differential equation system given in Eq 1 since it is expressed in terms of populations *n*_*i*_, but the EPR experiment observes signal intensities proportional to population differences *n*_*i*_ − *n*_*j*_ between the triplet sublevels *i* and *j*. Thus, only two initial values in terms of signal intensity at time *t*_DAF_ = 0 are given. The third value is calculated using the sum of signal intensities at the same point in time, which is proportional to a population difference of *n*_−1_ − *n*_+1_.

The deviation of the kinetic simulation from the experimental data is calculated as the reduced *χ*^2^-value of all temperatures and orientations. This procedure is repeated a given number of times with randomized initial parameters for every minimization. In general, 100 repetitions were suitable to approximate the global minimum adequately.

*Error Estimation* In order to determine the error of the best-fit relaxation parameters, a Monte Carlo simulation approach has been chosen to estimate the influence of random noise on the fitting procedure. For this purpose, the global minimum of all minimizations was used to generate complete sets of kinetic time traces (for each transition, orientation, and temperature), to which random noise was added to yield synthetic experimental data. The noise level was chosen to reflect the noise level of the experimental data. This way, at least 1000 data sets of overall 18 kinetic time traces were generated per haloanthracene, each affected differently by random noise. Subsequently, at least 1000 individual best-fit simulations were performed. The distribution of parameters is then visualized using histograms (cf. S7, S8, S9 and S10 Figs) and can be, in most cases, well described with Gaussian distributions. The width of these Gaussian distributions are estimates for the error in the best-fit parameter determination process with respect to the influence of random noise.

## Results and discussion

**Fig 2:**
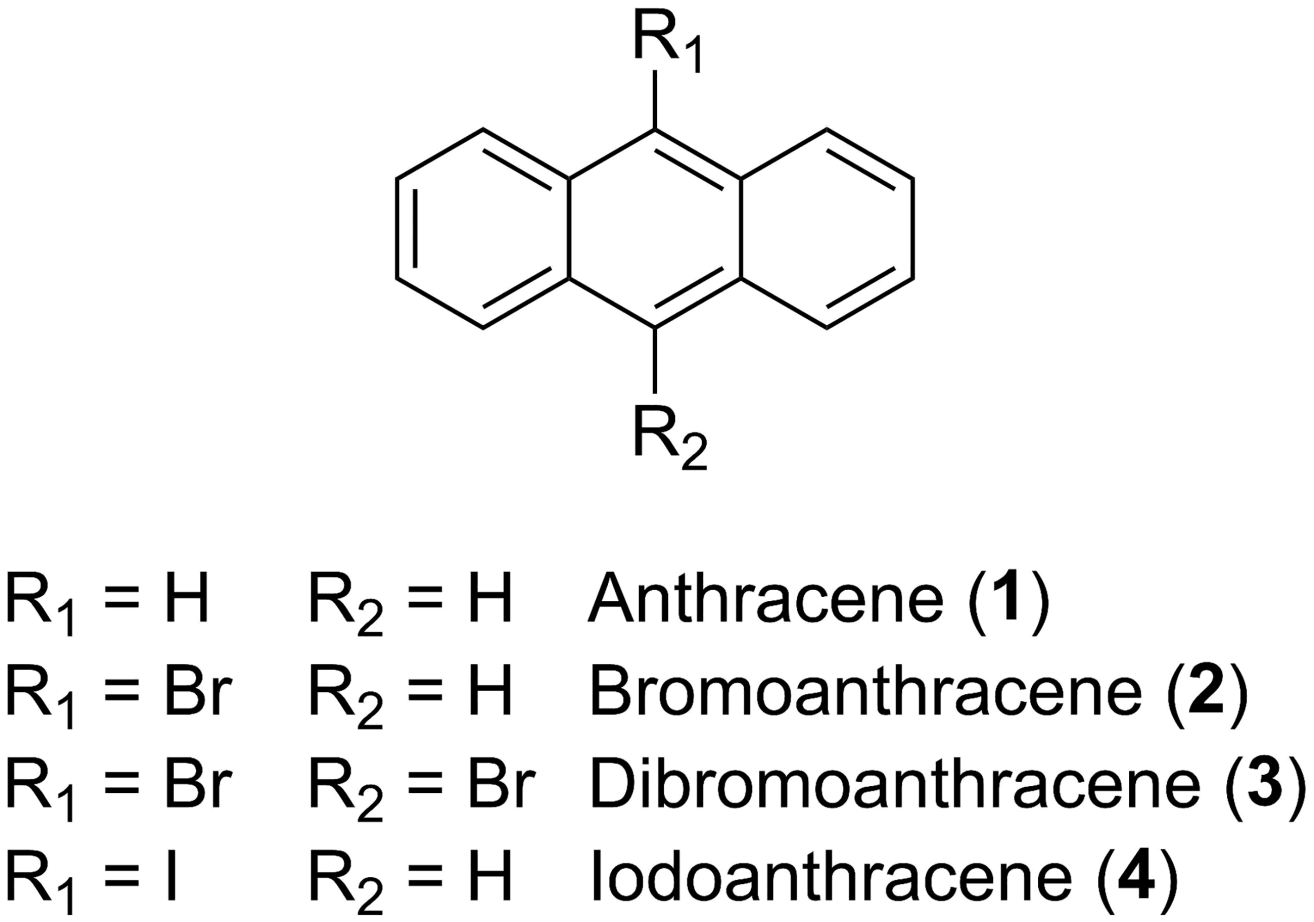
Structural formula of haloanthracenes.

**Fig 3:**
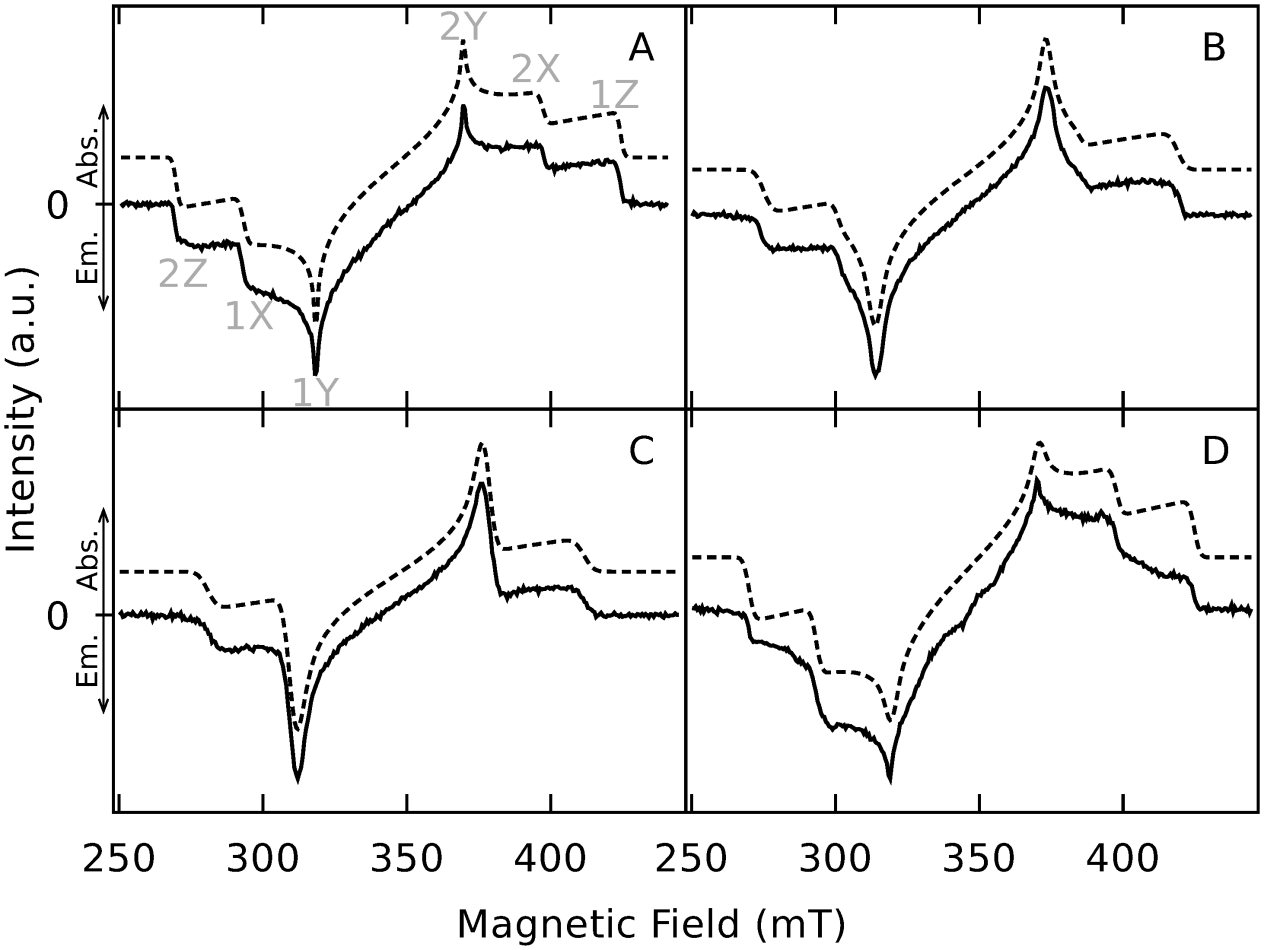
TR-EPR spectra of haloanthracenes. The TR-EPR spectra (solid lines) and corresponding spectral simulations (dashed, shifted vertically for better visibility). Absorption and emission are indicated by arrows at the left ordinate. A: Anthracene (**1**), B: Bromoanthracene (**2**), C: Dibromoanthracene (**3**), D: Iodoanthracene (**4**). Recorded at 20 K in X band. Relative sublevel populations and ZFS parameters of the simulations can be found in Table 1. For the full set of parameters, cf. S3 Table.

The EPR spectra of optically excited anthracenes **1** to **4** (Fig 2) have been recorded using magnetic field dependent direct detection of the TR-EPR signals. The EPR transients of each magnetic field are integrated for optimized SNR. Care was taken to not integrate up to too long delay times where the transients are already affected by SLR, thus maintaining correct information on OSP. Therefore, we do not analyze the TR-EPR data in terms of kinetics, but in terms of spectra. The TR-EPR spectra are shown in Fig 3. We were not able to detect EPR response from 9-chloroanthracene and 9-fluoroanthracene (not shown in Fig 2).

The ZFS parameters of **1** are reported in the literature [28], where they have been determined with continuous wave (cw) EPR spectroscopy of **1** in single crystals of biphenyl. It was found that–when the molecular axis system which diagonalizes the ZFS tensor is defined by X being the long axis, Y the short axis, and Z perpendicular to the molecular plane–the energetic order of the triplet substates is *T*_Z_ < *T*_Y_ < *T*_X_. This corresponds to positive *D* and negative *E*. Thus, in our work, we use the same axis system and signs for the ZFS parameters. The spectral positions corresponding to the canonical orientations are then assigned as indicated in Fig 3.

Since neither the ZFS nor the OSP change significantly within the series of haloanthracenes, we assume the same molecular axis system for the haloanthracenes **2** to **4** as used for **1**. This way, the spectra were simulated using EasySpin, a Matlab package [25]. The corresponding spin system’s relative sublevel populations and ZFS parameters are shown in Table 1. For the full set of parameters, cf. S3 Table. The reported values of the ZFS parameters of **1** [28] are in excellent agreement to our results.

The experimental spectra can be very well described by the spectral simulations overall, except for the intensities of the transitions of the canonical Z orientation of **4**. We would like to note that in the case of **3** the ZFS parameter *E* becomes very small. Most probably the electron withdrawing bromines lead to rather oblate spin density distribution (axial symmetry around and flattened along the Z axis) compared to **1**, where it is an elongated oblate (flattened along Z, but elongated along X).

**Fig 4:**
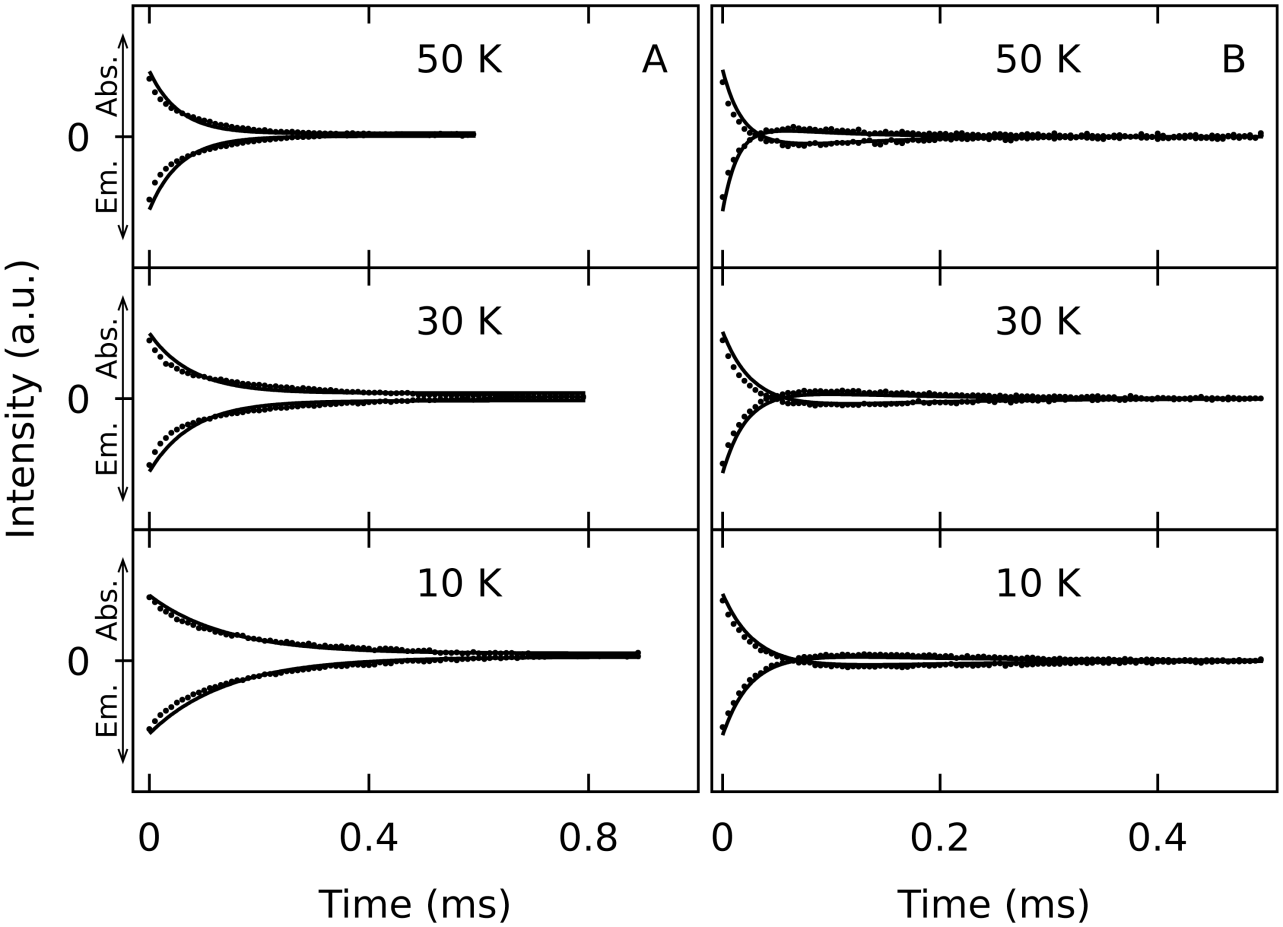
Exemplary ESE detected triplet kinetics of two haloanthracenes. A: Anthracene (**1**). B: Dibromoanthracene (**3**). The Hahn spin echo amplitude (dots) is recorded at different temperatures (50 K, 30 K, and 10 K, from top to bottom) in X band as a function of the delay time *t*_DAF_ after photoexcitation for both allowed EPR transitions on the Z canonical orientation, 1Z being emissive and 2Z being absorptive as indicated with arrows at the left ordinate. The parameters obtained from the best fit simulations (solid lines) are given in Table 2. Note the different time scales.

The triplet kinetics were recorded using time resolved ESE-EPR, varying *t*_DAF_, in the temperature range of 10 K to 50 K. Exemplary data is shown in Fig 4, for the full set of kinetic curves cf. S1, S2, S3 and S4 Figs. A small number of shots per point, that means a few laser excitations per time point, is used to avoid bleaching, which would influence the kinetic time traces. Bleaching only becomes apparent after several scans and is excluded from the data analysis. The temperature dependency is apparent in the case of **1** (Fig 4A), whereas it is not obvious in the case of **3** (Fig 4B). At the same time, as the only compound investigated, the latter shows polarization inversion of the initial OSP. Such behavior has been observed already for, e.g., pentacenes [23]. The polarization inversion corresponds to a fast depopulation of the *M*_S_ = 0 triplet substate compared to the depopulation the other two *M*_S_ = ±1 substates.

In general, the signal decay is faster at higher temperatures, which is as expected since SLR is generally faster at higher temperatures. Overall, the signal decays faster with increasing halide molecular weight, which might be due to fastened SLR or triplet decay. To further clarify this issue, the kinetic time traces have been simulated. The SLR times determined this way for every of the four anthracenes, the three temperatures, and the three canonical orientations are given in Table 2. The corresponding triplet life times are given in Table 3.

**Table 2:** SLR times of haloanthracenes obtained from best fit simulations of ESE EPR detected triplet kinetics, partially shown in Fig 4. For the full set of decay curves and their simulations, cf. S1, S2, S3 and S4 Figs. Since in the case of 3, the signals of the canonical X and Y direction can not be distinguished spectrally, the mutual SLR times are given in the X columns. The numbers in braces indicate the width of the distributions of the parameters, described in terms of 2*σ* of corresponding normal distributions, as obtained from Monte Carlo simulations.

		$w_{iX}^{-1}$		$w_{iY}^{-1}$		$w_{iZ}^{-1}$	
	*T*	*i* = 1	2	1	2	1	2
	K	μs	μs	μs	μs	μs	μs
**1**	50	101(29)	613 (239)	86(24)	549 (21)	187 (71)	151(49)
	30	162(45)	877 (361)	145(47)	1076(445)	277 (117)	206(79)
	10	389(150)	1637(596)	372(125)	8868(3525)	581(215)	349(122)
**2**	50	84(1)	115(1)	113(1)	93(1)	86(1)	50(1)
	30	144(1)	220(1)	221(1)	162(1)	188(1)	108(1)
	10	212(1)	413(2)	326(1)	248(1)	331(1)	173(1)
**3**	50	48(4)	151(34)			280(125)	39(25)
	30	67(6)	345(143)			1088(433)	91(46)
	10	87(10)	566(304)			1194(483)	148(90)
**4**	50	33(9)	55(31)	28(13)	32(20)	10(10)	10(10)
	30	47(13)	65(26)	73(18)	85(23)	10(10)	10(10)
	10	74(18)	209(41)	173(42)	173(33)	103(48)	94(38)

**Table 3:** Triplet life times of haloanthracenes obtained from best fit simulations of ESE EPR detected triplet kinetics, as exemplary shown in Fig 4. For the full set of decay curves and their simulations, cf. S1, S2, S3 and S4 Figs. Since in the case of 3, the signals of the canonical X and Y direction can not be distinguished spectrally, the mutual triplet life times are given in the X column. For 1, the triplet life time has been determined in a separate measurement (cf. S5 Fig), in good agreement to the value found in the literature [29] (≈ 40 ms), and was held constant. In the cases of 1 and 2 no triplet decay anisotropy was found, meaning that *k*_X_ = *k*_Y_ = *k*_Z_ = *k*. The numbers in braces indicate the width of the distributions of the parameters, described in terms of 2*σ* of corresponding normal distributions, as obtained from Monte Carlo simulations.

	$k_{X}^{-1}$ ms	$k_{Y}^{-1}$ ms	$k_{Z}^{-1}$ ms
**1**	33(3)	33(3)	33(3)
**2**	0,11(1)	0,11(1)	0,11(1)
**3**	1,4(6)	0,017(2)	4(2)
**4**	2,2(5)	0,035(2)	0,011(1)

**Fig 5:**
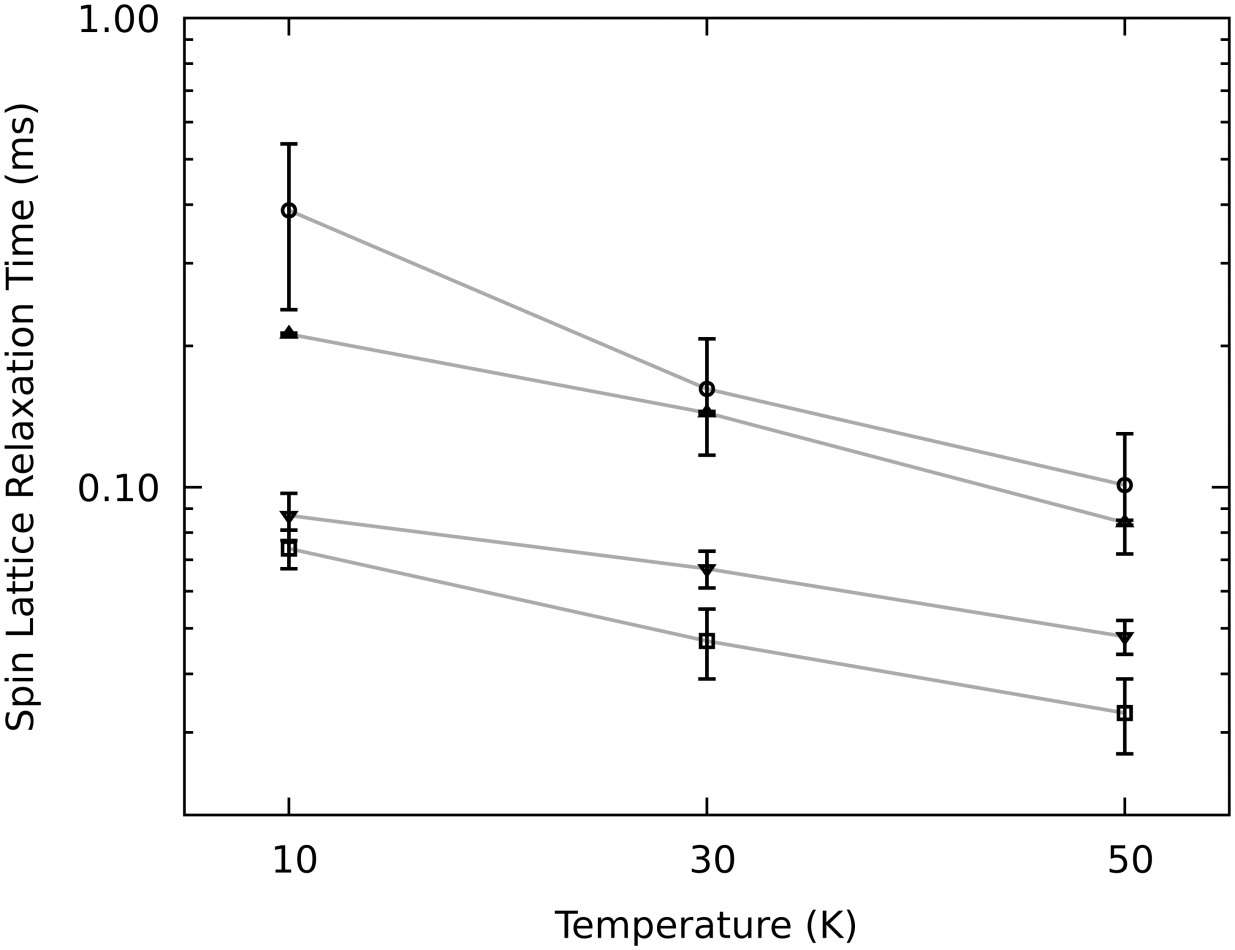
Semilogarithmic plot of the temperature dependency of SLR times of anthracenes and the effect of halogenation. SLR times determined through best fit simulations of the ESE detected triplet kinetics versus temperature. The SLR time as well as its dependency on the temperature decreases with increasing halide molecular weight from **1** (circles), **2** (triangles up), **3** (triangles down) and **4** (squares). Exemplary data shown for the 1X transition. For the full set of parameters of all transitions and orientations cf. Table 2.

As can be seen in Table 3, the triplet life time is shortened by two orders of magnitude when halides are introduced into the aromatic system. Within the series of haloanthracenes, the triplet life time further decreases with increasing molecular weight of the halide while becoming highly anisotropic with respect to the molecular axes. The effect upon the SLR is not seen as easily in this rather big data set of Table 2. Therefore, a subset of this data is illustrated in Fig 5.

Fig 5 exemplifies the SLR times for anthracenes **1** to **4** for all temperatures on the 1X transition. The SLR time is shortened significantly by the introduction of halides, further decreasing with increasing molecular weight of the halide.

## Conclusion

In order to tailor a given chromophore to suit the needs of its application, one has to address two issues. First of all, it is useful to have means to estimate the effect of chemical substitutions e.g. in terms of the heavy-atom effect. Second, a procedure to assess the kinetic parameters easily and efficiently is required. Experimentally, the latter is established already, while under several conditions the analysis still relies on approximations. [14] In this work, a procedure to simulate the kinetic data by numerically solving the exact differential equation system describing excited triplet state kinetics is described.

To illustrate an approach to the first issue, a systematic investigation of the heavy-atom effect in haloanthracenes is presented. It is found that halogenation of anthracenes in the 9,10-positions with bromine and iodine facilitates both, SLR as well as triplet decay. The triplet life time is shortened by two orders of magnitude by the introduction of bromine. Both, the SLR and triplet life time further decrease when a second bromine is added or when bromine is substituted with iodine.

## Supporting information

S1 Fig Kinetic time traces of 1 (black dots) with best-fit simulations (red lines). Measured at 50 K, 30 K, and 10 K (from top to bottom) on spectral components corresponding to the canonical orientations (X, Y, and Z, from left to right). Intensities are on scale and correspond to the actual spectral intensities.
(PDF)

S2 Fig Kinetic time traces of 2 (black dots) with best-fit simulations (red lines). Measured at 50 K, 30 K, and 10 K (from top to bottom) on spectral components corresponding to the canonical orientations (X, Y, and Z, from left to right). Intensities are on scale and correspond to the actual spectral intensities.
(PDF)

S3 Fig Kinetic time traces of 3 (black dots) with best-fit simulations (red lines). Measured at 50 K, 30 K, and 10 K (from top to bottom) on spectral components corresponding to the canonical orientations ((X, Y) and Z, from left to right). Intensities are on scale and correspond to the actual spectral intensities.
(PDF)

S4 Fig Kinetic time traces of 4 (black dots) with best-fit simulations (red lines). Measured at 50 K, 30 K, and 10 K (from top to bottom) on spectral components corresponding to the canonical orientations (X, Y, and Z, from left to right). Intensities are on scale and correspond to the actual spectral intensities.
(PDF)

S5 Fig Kinetic time traces of 1 with low time resolution. Measured at 50 K (red circles), 30 K (blue squares), and 10 K (green diamonds) on the 2Y transition. The raw data is baseline corrected, normalized, and plotted semi logarithmic for easy comparison.
(PDF)

S6 Fig Normalized UV/Vis absorption spectra of all anthracenes in toluene. Measured at room temperature. Anthracene (**1**) and Anthracene-*d*10 (**0**) are virtually identical. Vertical lines indicate the wavelength used for triplet excitation. The spectra exhibit bathochromic shift with increasing halide molecular weight.
(PDF)

S7 Fig Histograms of the distributions of parameters of individual minimizations performed on synthetic data with varying influence of white noise (blue bars) with a Gaussian fit (red lines) for 1. (PNG)

S8 Fig Histograms of the distributions of parameters of individual minimizations performed on synthetic data with varying influence of white noise (blue bars) with a Gaussian fit (red lines) for 2. (PNG)

S9 Fig Histograms of the distributions of parameters of individual minimizations performed on synthetic data with varying influence of white noise (blue bars) with a Gaussian fit (red lines) for 3. (PNG)

S10 Fig Histograms of the distributions of parameters of individual minimizations performed on synthetic data with varying influence of white noise (blue bars) with a Gaussian fit (red lines) for 4. (PNG)

S1 Table Numbers of averages done in the TR-EPR experiments. (HTML)

S2 Table Shots per point, number of scans and number of points used in the ESE EPR experiments. In case of **1**, different number of points were used for different temperatures. They are given in the following sequence: 10 K, 30 K, and 50 K.
(HTML)

S3 Table Residual linewidth (FWHM) in MHz of the anisotropic broadening due to unresolved hyperfine couplings and g-tensors found by best-fit spectral simulations. (HTML)
