# Supplementary figures and images for: Heavy-atom effect on optically excited triplet state kinetics
Source: PLoS One. 2017 Nov 20;12(11):e0184239. doi: 10.1371/journal.pone.0184239 (PMC5695852; doi:10.1371/journal.pone.0184239)

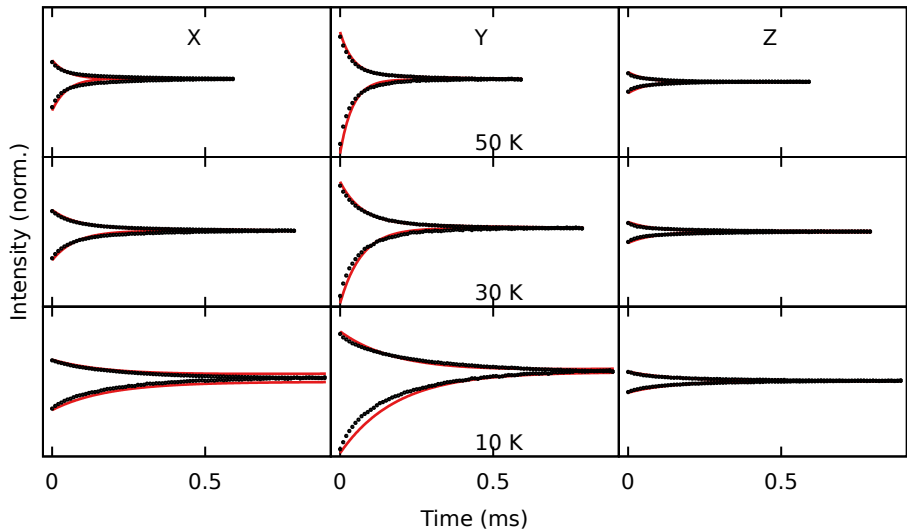

Supplement: S1 Fig — Measured at 50 K, 30 K, and 10 K (from top to bottom) on spectral components corresponding to the canonical orientations (X, Y, and Z, from left to right). Intensities are on scale and correspond to the actual spectral intensities. (PDF) [file pone.0184239.s001.pdf]

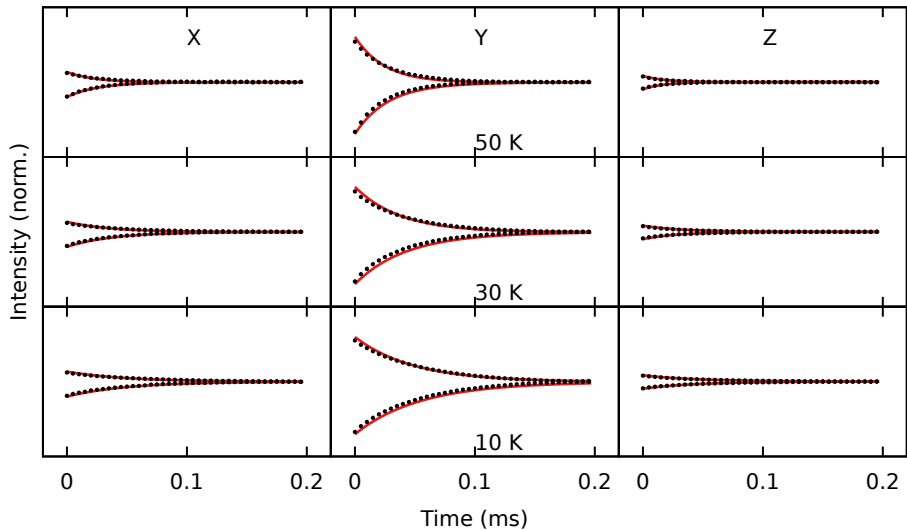

Supplement: S2 Fig — Measured at 50 K, 30 K, and 10 K (from top to bottom) on spectral components corresponding to the canonical orientations (X, Y, and Z, from left to right). Intensities are on scale and correspond to the actual spectral intensities. (PDF) [file pone.0184239.s002.pdf]

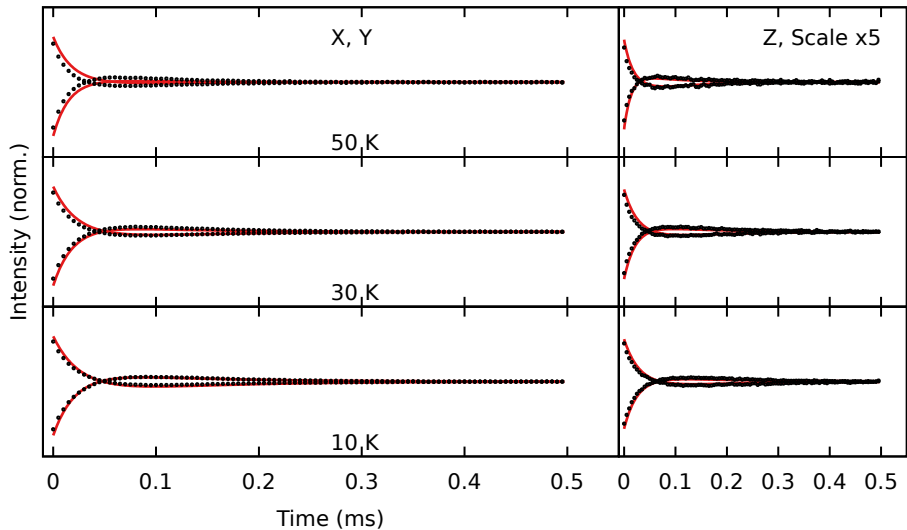

Supplement: S3 Fig — Measured at 50 K, 30 K, and 10 K (from top to bottom) on spectral components corresponding to the canonical orientations ((X, Y) and Z, from left to right). Intensities are on scale and correspond to the actual spectral intensities. (PDF) [file pone.0184239.s003.pdf]

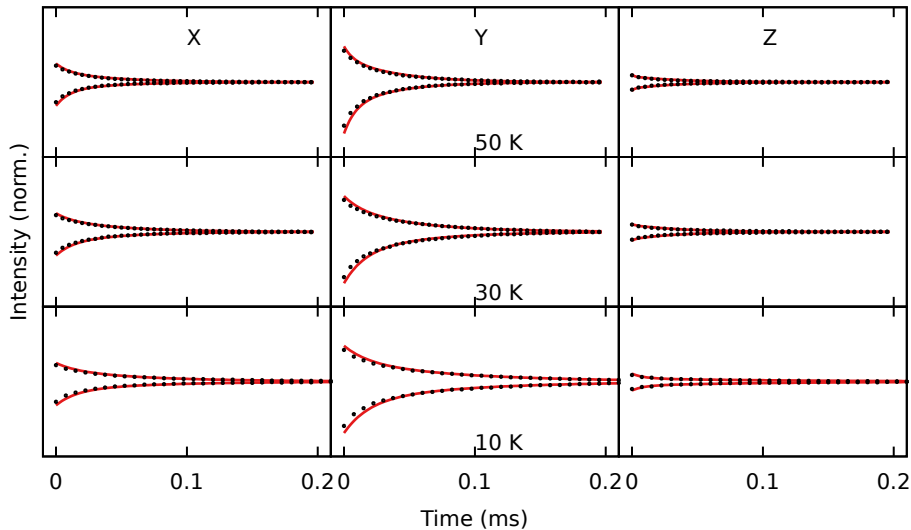

Supplement: S4 Fig — Measured at 50 K, 30 K, and 10 K (from top to bottom) on spectral components corresponding to the canonical orientations (X, Y, and Z, from left to right). Intensities are on scale and correspond to the actual spectral intensities. (PDF) [file pone.0184239.s004.pdf]

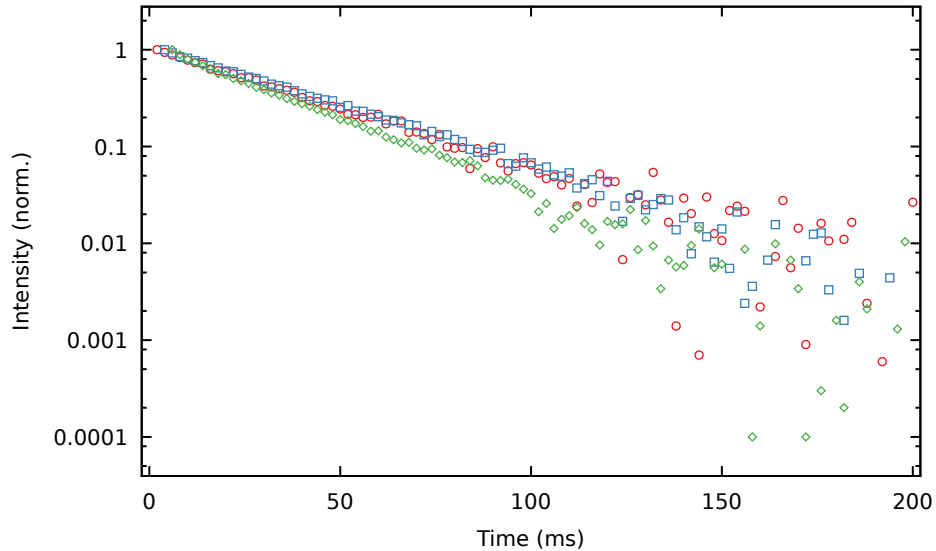

Supplement: S5 Fig — Measured at 50 K (red circles), 30 K (blue squares), and 10 K (green diamonds) on the 2Y transition. The raw data is baseline corrected, normalized, and plotted semi logarithmic for easy comparison. (PDF) [file pone.0184239.s005.pdf]

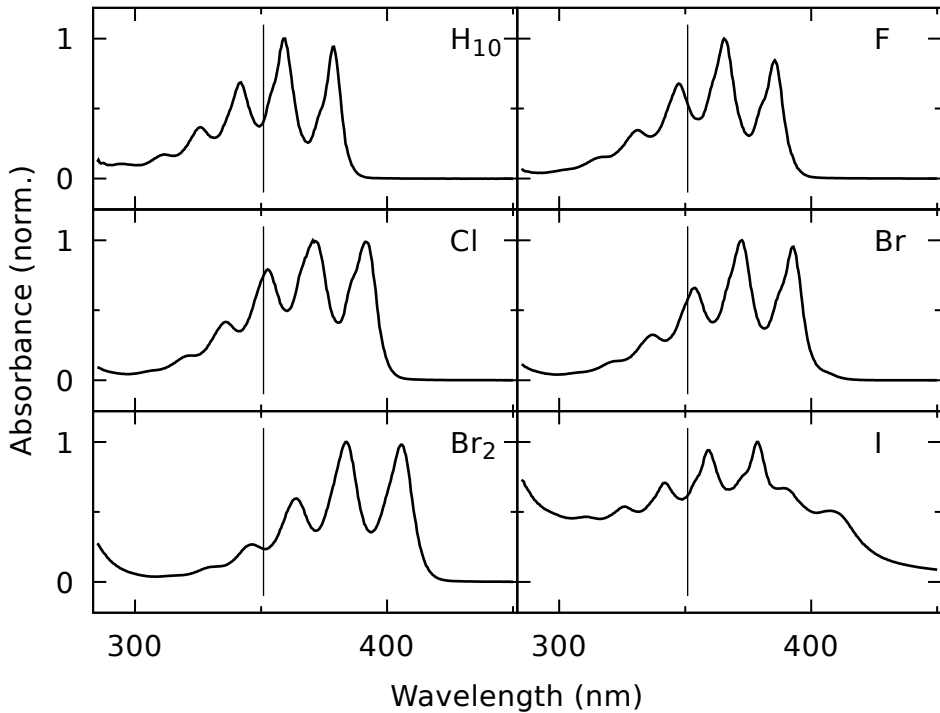

Supplement: S6 Fig — Measured at room temperature. Anthracene (1) and Anthracene-d10 (0) are virtually identical. Vertical lines indicate the wavelength used for triplet excitation. The spectra exhibit bathochromic shift with increasing halide molecular weight. (PDF) [file pone.0184239.s006.pdf]
