# Supplementary material for: Heavy-atom effect on optically excited triplet state kinetics
Source: PLoS One. 2017 Nov 20;12(11):e0184239. doi: 10.1371/journal.pone.0184239 (PMC5695852; doi:10.1371/journal.pone.0184239)
Supplement: S1 Table — (HTML) [file pone.0184239.s011.html]

xml version="1.0" encoding="UTF-8"?
S1 Table 

|  | Transients per Point | Number of Scans |  |
| 1 | 5 | 18 |  |
| 2 | 6 | 4 |  |
| 3 | 6 | 38 |  |
| 4 | 6 | 11 |  |
|  |  |  |  |
|  |  |  |  |
