# Supplementary material for: Heavy-atom effect on optically excited triplet state kinetics
Source: PLoS One. 2017 Nov 20;12(11):e0184239. doi: 10.1371/journal.pone.0184239 (PMC5695852; doi:10.1371/journal.pone.0184239)
Supplement: S2 Table — In case of 1, different number of points were used for different temperatures. They are given in the following sequence: 10 K, 30 K, and 50 K. (HTML) [file pone.0184239.s012.html]

xml version="1.0" encoding="UTF-8"?
S2 Table 

|  | Shots per Point | Number of Scans | Number of Points |
| 1 | 5 | 18 | 450 / 400 / 300 |
| 2 | 6 | 4 | 200 |
| 3 | 6 | 38 | 500 |
| 4 | 6 | 11 | 200 |
