# Supplementary material for: Heavy-atom effect on optically excited triplet state kinetics
Source: PLoS One. 2017 Nov 20;12(11):e0184239. doi: 10.1371/journal.pone.0184239 (PMC5695852; doi:10.1371/journal.pone.0184239)
Supplement: S3 Table — (HTML) [file pone.0184239.s013.html]

xml version="1.0" encoding="UTF-8"?
S3 Table 

|  |  | g |  |  | Hstrain |  |
|  | XX | YY | ZZ | X | Y | Z |
| 1 | 2.0039 | 2.0060 | 2.0046 | 86 | 38 | 98 |
| 2 | 2.0093 | 2.0110 | 2.0069 | 45 | 101 | 152 |
| 3 | 2.0158 | 2.0152 | 2.0152 | 112 | 63 | 242 |
| 4 | 2.0039 | 2.0038 | 2.0036 | 133 | 50 | 100 |
